# Dynamic gut microbiota shift in chronic liver disease progression: machine learning models and the E/Er ratio as a predictive biomarker

**DOI:** 10.1186/s12967-026-08499-y

**Published:** 2026-06-25

**Authors:** Dai Mengting, Ji Zhaoyang, Dong Jingjing, Wu Jianbo, Ye Tongtong, Zhou Linling, Qin Jiaying, Luo Jialu, Chen Ping, Xu Mingzhi

**Affiliations:** 1 Key Laboratory of Artificial Organs and Computational Medicine of Zhejiang Province, Hangzhou, Zhejiang China; 2 Department of General Medicine, Hangzhou Institute of Medicine (HIM), Zhejiang Cancer Hospital, Chinese Academy of Sciences, Hangzhou, 310022 Zhejiang China; 3 Department of Endocrinology and Metabolic Disease, Shulan (Hangzhou) Hospital Affiliated to Zhejiang Shuren University Shulan International Medical College, Hangzhou, Zhejiang China; 4 The Second Clinical Medical College of Zhejiang, Chinese Medical University, Hangzhou, 310053 Zhejiang China; 5 Wenzhou Medical University, Wenzhou, Zhejiang China; 6 Department of Infectious Diseases, Shulan (Hangzhou) Hospital Affiliated to Zhejiang Shuren University Shulan International Medical College, Hangzhou, Zhejiang China

**Keywords:** Gut-liver axis, Chronic liver disease, Acute-on-chronic liver failure, Gut microbiota, Machine learning, *Enterococcus*, *Eubacterium rectale*, E/Er Ratio (*Enterococcus/Eubacterium rectale Ratio*), Model Construction, Cutoff

## Abstract

**Background:**

Chronic liver disease (CLD) and its progression to compensated cirrhosis (CC), decompensated cirrhosis (DC), and acute-on-chronic liver failure (ACLF) represent a global health crisis with high mortality. The “gut-liver axis” plays a pivotal role in this progression, yet a systematic characterization of the dynamic microbial evolution across the entire disease spectrum remains elusive. We aimed to systematically characterize the gut microbiota’s dynamic evolution across the spectrum of chronic liver disease and to develop machine-learning models for predicting its critical transitions.

**Methods:**

In this retrospective cohort study, 947 patients with liver disease (CLD, *n* = 464; CC, *n* = 159; DC, *n* = 260; ACLF, *n* = 64) were enrolled. The relative abundances of 10 dominant gut microbiota—including *Enterococcus*,* Lactobacillus*,* Clostridium leptum*,* Clostridium butyricum*,* Eubacterium rectale*,* Faecalibacterium prausnitzii*,* Bacteroides*,* Enterobacterium*,* Bifidobacterium*, and *Atopobium cluster*—were quantified via qPCR. Random Forest (RF) models were constructed to predict transitions between disease stages. The discriminatory efficacy of the *Enterococcus/Eubacterium rectale* Ratio (E/Er Ratio) was further evaluated using Receiver Operating Characteristic (ROC) analysis.

**Results:**

Four distinct dynamic evolutionary patterns were identified. The pro-inflammatory/anti-inflammatory ratio increased 5.4-fold from CLD to ACLF. Random Forest models accurately predicted all disease transitions. The model for the DC-to-ACLF transition performed best (Area Under the Curve, AUC = 0.961), with clinical parameters (PT, TBil) being the strongest predictors. While the addition of microbial features yielded a modest incremental gain in AUC for the late-stage transition, indices such as the Specific-Butyrate-to-Total ratio were identified as key features, providing critical biological insights into the systemic failure of the gut-liver axis. The E/Er Ratio further served as a robust, non-invasive marker for identifying critical disease turning points.

**Conclusion:**

Liver disease progression is characterized by a systematic shift in the gut microbiota from an anti-inflammatory, butyrate-rich state to a pro-inflammatory, pathogen-dominant environment. The integrated RF models and the E/Er Ratio provide a powerful, non-invasive framework for the early prediction and risk stratification of chronic liver disease progression, offering potential targets for gut-centered therapeutic interventions.

**Supplementary Information:**

The online version contains supplementary material available at https://doi.org/10.1186/s12967-026-08499-y.

## Introduction

Chronic liver disease (CLD) and its progression to compensated cirrhosis (CC), decompensated cirrhosis (DC), and acute-on-chronic liver failure (ACLF) collectively impose a significant global health burden, characterized by high morbidity and mortality [1, 2]. Liver disease progression is a dynamic and continuous process, with early stages often lacking typical clinical symptoms [3]. This frequently causes many patients to miss the optimal window for therapeutic intervention. Therefore, a critical challenge in current hepatology research is the identification of non-invasive biomarkers capable of accurately predicting the turning points of disease progression at an early stage, thereby facilitating the development of effective early intervention strategies.

### The gut-liver axis and microbial dysbiosis in liver disease

Against this backdrop, the emergence of the “Gut-Liver Axis” theory has provided a novel perspective for understanding liver disease progression [4, 5]. The liver and the gut are anatomically and functionally intertwined, communicating bidirectionally via the portal vein, the biliary system, and systemic circulation. Accumulating evidence highlights the central role of the gut microbiota and their metabolites in maintaining liver health.

When the intestinal microbial ecosystem is imbalanced, a condition termed dysbiosis occurs [6]. This dysbiosis impairs the intestinal barrier function, leading to the translocation of microbial products, including pathogen-associated molecular patterns (PAMPs), to the liver. These products, in turn, activate Toll-like receptors (e.g., TLR4) on immune cells such as Kupffer cells, triggering a cascade of inflammatory responses that drive the processes of liver injury, fibrosis, and ultimately, liver failure [7, 8]. This shift in understanding—from the gut being affected by liver disease (“liver disease-gastro-caused”) to the gut being a therapeutic target for liver disease (“liver disease-gastro-treated”)—marks the intestinal microecology as a potential new focus for therapeutic intervention in liver diseases.

### Dynamic changes in key gut microbiota during liver disease progression

Numerous studies have elucidated the dynamic evolutionary patterns of the gut microbiota during the progression of liver disease. In the stages of Metabolic Dysfunction-Associated Steatotic Liver Disease (MASLD) and cirrhosis, a progressive depletion of butyrate-producing bacteria with anti-inflammatory properties, such as *Faecalibacterium prausnitzii*, has been observed [9]. As the disease advances to the decompensated stage, this imbalance is often characterized by an abnormal expansion of pro-inflammatory microbiota, such as *Enterococcus*, alongside a further reduction in anti-inflammatory commensals—a shift closely linked to intestinal barrier impairment and the onset of systemic inflammation [10].

Recent research further indicates that in patients with ACLF and corresponding murine models, there is a significant reduction in both the abundance of the gut microbe Megamonas funiformis and the levels of its key metabolite, panose. Experimental evidence suggests that panose supplementation can delay the progression of ACLF by mitigating oxidative stress and enhancing intestinal barrier function [11]. Additionally, the profound depletion of *Bifidobacterium* has been identified as a significant microbial hallmark of the progression from cirrhosis to hepatocellular carcinoma (HCC) [12].

### Current gaps and the role of machine learning

Despite these foundational discoveries, existing research is largely limited to cross-sectional comparisons of microbial composition across different disease stages. However, a systematic, longitudinal characterization of microbial dynamics across the entire disease continuum is still lacking. Crucially, the transformation of this dynamic pattern into a practical tool capable of accurately predicting the progression of CLD to the critical turning points of CC, DC, and ACLF remains an area of unmet need.

Machine learning algorithms, particularly the Random Forest model, are adept at efficiently processing high-throughput microbial data and identifying the most predictive features from complex biological signals. This approach has proven highly advantageous in disease diagnosis and classification.

### Objectives and proposed workflow

**Therefore**,** this study aims to**:

1. **Systematically delineate the dynamic change spectrum** of the 10 dominant gut microbiota throughout the entire course of chronic liver disease.
2. **Construct high-accuracy Random Forest predictive models** for the three critical transition points: CLD → CC, CC → DC, and DC → ACLF.
3. **Screen and validate core**,** universally applicable microbial biomarkers** across multiple stages derived from the predictive models.

**Fig. 1:**
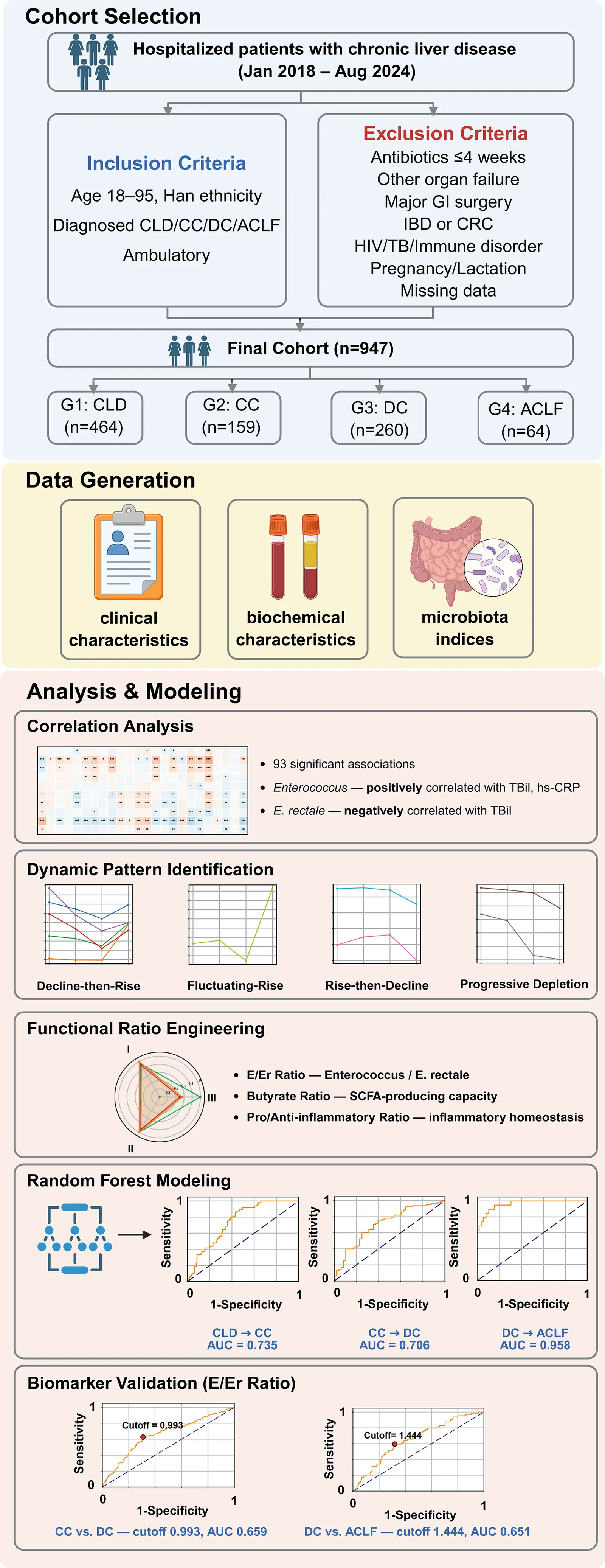
Schematic overview of the study design and analytical workflow. The flowchart details patient screening, inclusion and exclusion criteria, cohort stratification (CLD, *n* = 464; CC, *n* = 159; DC, *n* = 260; ACLF, *n* = 64), data acquisition (clinical parameters and qPCR-based gut microbiota profiling), and the analytical workflow from correlation analysis and dynamic pattern identification to Random Forest model construction and biomarker validation

This research uniquely integrates the dynamic evolutionary pattern of the gut microbiota with machine learning prediction models, striving to establish a non-invasive early warning system that spans the entire continuum of chronic liver disease. A schematic overview of the study workflow is presented in Fig. 1. Our findings are expected to not only offer new insights into the microbial mechanisms of liver disease progression but also to provide powerful translational tools for the realization of early and precise intervention in liver diseases.

## Materials and methods

### Study design and patient cohort

This study was a retrospective cohort analysis that enrolled a total of 947 hospitalized patients with liver disease admitted between January 2018 and August 2024 at a comprehensive Grade A Tertiary Hospital in Zhejiang Province, China. All fecal and blood samples were collected at the time of hospital admission (baseline) for each patient. This study employed a cross-sectional design, with inter-group comparisons serving as proxies for disease progression.

#### Diagnostic criteria

Patients were categorized into four distinct disease stages based on established clinical and radiological criteria:

##### CLD

Persistent liver injury caused by any chronic etiology, such as Hepatitis B Virus (HBV), Hepatitis C Virus (HCV), alcoholic liver disease (ALD), or non-alcoholic fatty liver disease (NAFLD), but without radiological or clinical evidence of cirrhosis.

##### CC

Cirrhosis confirmed by liver histology or a combination of clinical, radiological (e.g., changes in liver morphology, splenomegaly, widening of the portal vein), and laboratory findings (e.g., thrombocytopenia, reduced albumin), with no previous history of decompensating events such as ascites, hepatic encephalopathy (HE), or variceal bleeding.

##### DC

Confirmed cirrhosis with the occurrence of definitive decompensating events, including but not limited to: ascites (clinically or radiologically confirmed), hepatic encephalopathy or esophageal/gastric variceal bleeding.

##### ACLF

Acute deterioration of liver function in a patient with underlying chronic liver disease (predominantly cirrhosis), complicated by organ failure. The diagnosis adhered to the Asian Pacific Association for the Study of the Liver (APASL) criteria, defined by the presence of jaundice (total bilirubin≥5 mg/dL) and coagulopathy (INR ≥ 1.5 or Prothrombin Activity < 40%), followed by ascites and/or hepatic encephalopathy within four weeks.

#### Inclusion criteria

Patients were included if they met the following criteria:

1. Adults aged 18–95 years, regardless of sex, of Han ethnicity.
2. Able to communicate, consume food, and ambulate normally.
3. Met the diagnostic criteria for any of the aforementioned disease stages.

#### Exclusion criteria

Patients were excluded if they met any of the following conditions:

1. Concomitant end-stage functional failure of other vital organs (e.g., heart, lung, kidney).
2. Co-existing Human Immunodeficiency Virus (HIV) infection, active pulmonary tuberculosis, or other severe systemic immune disorders.
3. Use of antibiotics, prebiotics, probiotics, or immunosuppressants within the last four weeks, which could significantly influence gut microbiota composition.
4. Prior history of major gastrointestinal surgery.
5. Co-existing inflammatory bowel disease (IBD), colorectal cancer, or other known organic intestinal diseases.
6. Pregnant or lactating women.

### Data collection

#### Clinical parameters

The available laboratory results collected during patient admission or follow-up were retrospectively extracted from the Electronic Medical Record (EMR) system, including:

- **Liver Function**: Total bilirubin, direct bilirubin, indirect bilirubin, albumin, alanine aminotransferase (ALT), aspartate aminotransferase (AST), γ-glutamyl transferase (γ-GT), and alkaline phosphatase (ALP).
- **Coagulation Function**: Prothrombin time (PT), activated partial thromboplastin time (APTT), thrombin time (TT), fibrinogen, and D-dimer.
- **Inflammatory Markers**: C-reactive protein (CRP), high-sensitivity C-reactive protein (hs-CRP), white blood cell count (WBC), neutrophil count, lymphocyte count, and platelet count.
- **Metabolic and Nutritional Indices**: Total cholesterol (TC), triglycerides (TG), high-density lipoprotein (HDL), low-density lipoprotein (LDL), uric acid (UA), blood urea nitrogen (BUN), creatinine (Cr), estimated glomerular filtration rate (eGFR), thyroid function tests (T3, T4, fT3, fT4, TSH), and electrolytes (Na, K, Cl, Ca, P).
- **Composite Inflammatory Indices**: Systemic Immune-Inflammation Index (SII), Neutrophil-to-Lymphocyte Ratio (NLR), Platelet-to-Lymphocyte Ratio (PLR), and Neutrophil Percentage-to-Albumin Ratio (NPAR).

#### qPCR quantification of gut microbiota

Mid-posterior fecal samples were expelled into a sterile container, and 2.0 g of fresh feces were collected from the medial middle portion using a sterile spoon into a disposable sterile stool collector. Samples were immediately transported to the laboratory, aliquoted into 10 centrifuge tubes (0.2 g each), and stored at -80 °C within 30 min of collection.

Each centrifuge tube received 1.3 mL of InhibitEX Buffer and a glass bead (2 mm diameter, 0.3 g). Samples were homogenized on a vortex oscillator at 5000 rpm for two 30-second cycles, followed by centrifugation at 15,000 rpm for 3 min twice. DNA was extracted using the QIAamp Fast DNA Stool Mini Kit following the manufacturer’s instructions.

Absolute quantification of 10 dominant gut microbiota was performed by quantitative real-time PCR (qPCR) on an ABI ViiA7 system (Applied Biosystems, USA). The targeted microbiota included *Atopobium cluster*, *Clostridium leptum*, *Lactobacillus*, *Enterobacterium*, *Bacteroides*, *Enterococcus*, *F. prausnitzii*, *Bifidobacterium*, *Eubacterium rectale* and *Clostridium butyricum*. The 10 dominant gut microbiota were selected based on: (i) established relevance to the gut-liver axis, (ii) functional representation of major short-chain fatty acid (SCFA) producers, opportunistic pathogens, and dominant commensals, and (iii) clinical translatability of well-validated qPCR assays. Primer sequences, annealing temperatures, and references are provided in Supplementary Table S1.

The 20 µL PCR reaction mixture contained: 10 µL SYBR Green PCR Master Mix, 0.4 µL forward primer, 0.4 µL reverse primer, 2 µL DNA template, and 7.2 µL RNase-free H₂O. Thermal cycling conditions were: pre-denaturation at 95 °C for 3 min, followed by 40 cycles of denaturation at 95 °C for 30 s, annealing at 58 °C for 40 s, and extension at 72 °C for 5 min. Fluorescence intensity was measured for 10 s at the end of each cycle to avoid interference from secondary structure and primer dimers. The specificity of each primer set was confirmed by melting curve analysis (Supplementary Fig. S1). The initial copy number of the target gene in each sample was determined against standard curves, with each sample run in triplicate wells. All abundances were normalized to log₁₀ copies per gram of feces.

### Data preprocessing

Missing values in clinical parameters were imputed using the median value of the respective variable across all patients. Microbiota abundance data were normalized using sum-standardization before log transformation. Categorical variables were processed using label encoding, while continuous variables were standardized using Z-score normalization prior to their application in machine learning models.

### Calculation of microbiome-derived ratios

To quantify functional shifts in the gut ecosystem, the following indices were calculated:

1. E/Er Ratio = Enterococcus / Eubacterium rectale.
2. Pro-inflammatory/Anti-inflammatory Ratio = (Enterococcus + Enterobacterium) / (Lactobacillus + Bifidobacterium + C. butyricum + C. leptum + E. rectale + F. prausnitzii).
3. Butyrate-Pro Ratio = (E. rectale + F. prausnitzii + C. leptum + C. butyricum) / (Enterococcus + Enterobacterium).
4. Butyrate-Total Ratio = (E. rectale + F. prausnitzii + C. leptum + C. butyricum) / Total bacterial load.
5. Specific-Butyrate-Total Ratio = (E. rectale + F. prausnitzii) / Total bacterial load.
6. Specific-Butyrate-Pro Ratio = (E. rectale + F. prausnitzii) / (Enterococcus + Enterobacterium).

For the radar chart displaying multi‑dimensional microbial indices (Fig. 4D), the three selected ratios were further transformed and normalized to a 0–1 scale using Min–Max normalization across the four disease stages, with higher values representing a more beneficial microbial profile:

A. Normalized Butyrate Ratio = (Butyrate‑Total Ratio – min) / (max – min).
B. Normalized Anti‑inflammatory Balance = 1 – (Pro‑inflammatory/Anti‑inflammatory Ratio – min) / (max – min).
C. Normalized Eubiosis Index = 1 – (E/Er Ratio – min) / (max – min).

## Statistical analysis

### Correlation analysis

The associations between the abundance of the 10 specified gut microbiota and clinical parameters were evaluated using the Spearman’s rank correlation coefficient, a non-parametric method robust against deviations from normality. To address the issue of false positives arising from multiple hypothesis testing, two stringent correction methods were implemented: the Bonferroni correction to control the family-wise error rate (FWER) and the **Benjamini-Hochberg False Discovery Rate (FDR) correction**.

**Fig. 2:**
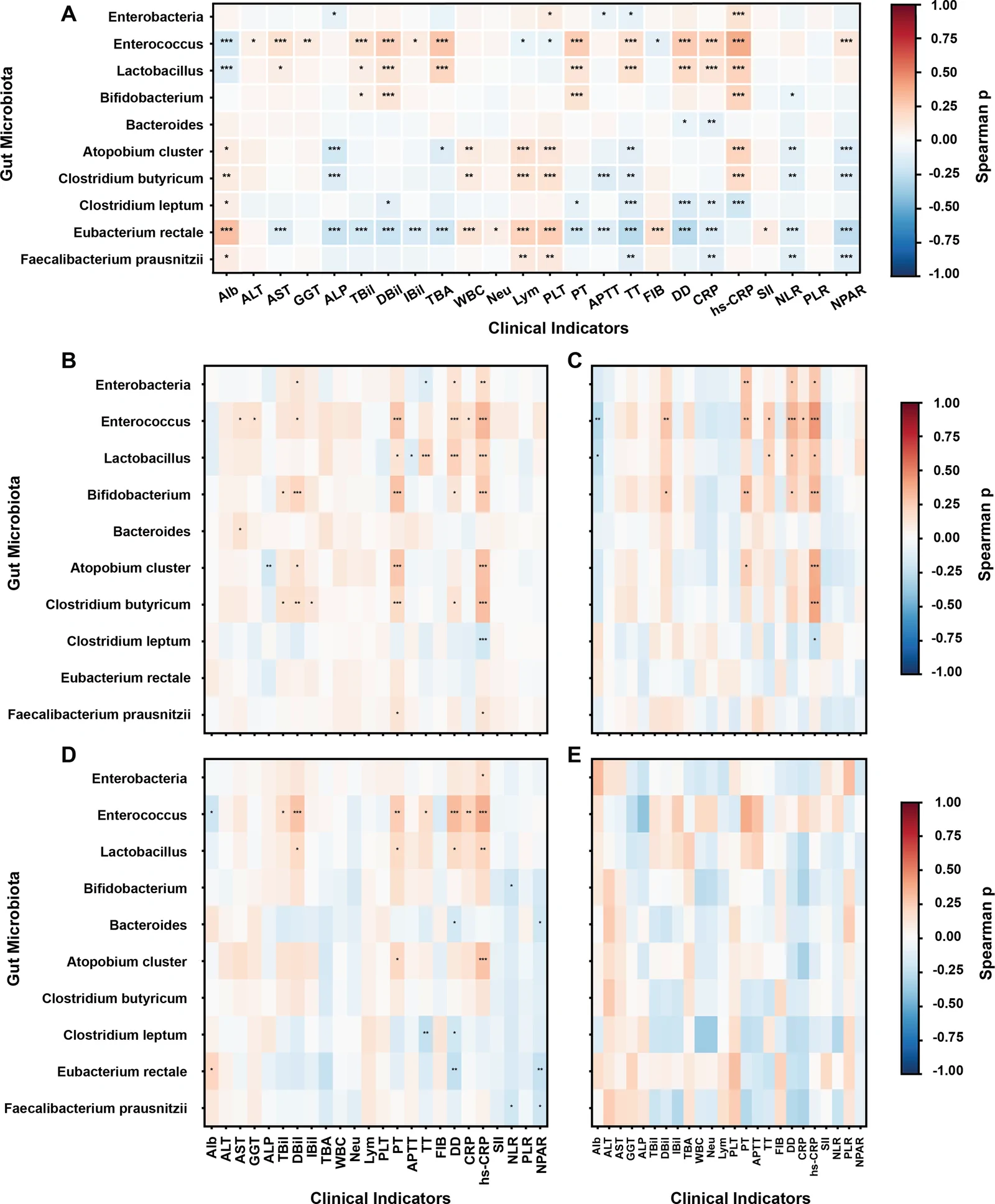
Heatmap analysis of clinical indices and gut microbiota across liver disease stages. (**A**) Overall Correlation Heatmap: This panel displays the Spearman correlation coefficients between the relative abundance of 10 dominant gut microbiota (y-axis) and various clinical biochemical and inflammatory parameters (x-axis) across the entire patient cohort. (**B–E**) Stage-Specific Correlation Heatmaps: These panels illustrate the specific correlation patterns within each disease stage: (**B**) CLD, (**C**) CC, (**D**) DC, and (**E**) ACLF. The 24 core parameters displayed represent key indicators of hepatic function, coagulation, and systemic inflammation. A comprehensive analysis of all 78 available clinical parameters is provided in Supplementary Table S3. Color Gradient: Correlation coefficients are represented by a color gradient, where red indicates a positive correlation and blue indicates a negative correlation. Statistical Significance: Asterisks denote levels of statistical significance: *P < 0.05, **P < 0.01, and ***P < 0.001. All *P*-values were adjusted for multiple comparisons using the False Discovery Rate (FDR) method

The resulting correlation matrix was visualized using a hierarchical clustering heatmap. Statistical significance was determined using a two-tailed *P*-value < 0.05 after applying the multiple testing corrections. For the primary heatmap (Fig. 2), a set of 24 core clinical parameters was selected to focus on three pathophysiological domains: hepatic function, coagulation, and systemic inflammation. A comprehensive analysis of all 78 clinical parameters is provided in Supplementary Table S3.

### Model construction and evaluation

#### Random forest classifier

##### Model construction

A Random Forest (RF) classifier was employed for disease progression prediction (CLD→CC, CC→DC, and DC→ACLF). The model was constructed using 1,000 decision trees. To handle potential class imbalance within the dataset, balanced class weights were set. The maximum depth of the trees was restricted to mitigate the risk of overfitting.

##### Feature selection

The RF algorithm was first used to rank the importance of the microbial features. We employed a forward selection strategy: microbial features were sequentially added based on their importance, and the optimal feature set was determined by maximizing the 5-fold cross-validated Area Under the Curve (AUC).

#### Dataset partition and model evaluation

The overall dataset was randomly split into a training set and a test set at a 7:3 ratio, utilizing stratified sampling to ensure that the proportions of cases and controls were maintained across both sets. Model performance was primarily assessed by the Area Under the Receiver Operating Characteristic Curve (AUC), along with secondary metrics including Accuracy, Sensitivity, and Specificity. The dataset was randomly shuffled prior to splitting using the default ‘shuffle=True’ parameter in ‘train_test_split’, with a fixed random seed (‘random_state = 42’) and stratified splitting (‘stratify = y’) to preserve class proportions.

#### Software tools

All statistical analyses and machine learning procedures were performed using Python 3.8. The primary libraries utilized included scikit-learn for machine learning, pandas for data manipulation, and scipy and statsmodels for statistical tests.

## Results

### Clinical characteristics of the study cohort

This study ultimately included a total of 947 patients categorized into four distinct groups: CLD (G1, *n* = 464), CC (G2, *n* = 159), DC (G3, *n* = 260), and ACLF (G4, *n* = 64). Significant statistical differences (*P* < 0.05) were observed across all groups in terms of age, gender distribution, and nearly all measured clinical, biochemical, and gut microbial indices, confirming the distinct pathophysiological features across the different stages of liver disease (as detailed in Tables 1 and 2).

As the disease progressed from CLD to ACLF, the study cohort exhibited a characteristic pattern of escalating hepatic dysfunction, synthetic failure, intensified systemic inflammation, and metabolic derangement.

**Table 1:** Demographic, clinical, and biochemical characteristics

Variable	CLD (*n* = 464)	CC (*n* = 159)	DC (*n* = 260)	ACLF (*n* = 64)	*P*
**[Demographics]**					
Age	51 (42,59)	54 (47,61)	55 (48,63)	53 (46,59)	< 0.001
Male	289 (62.3%)	97 (61.0%)	178 (68.5%)	51 (79.7%)	0.018
Female	175 (37.7%)	62 (39.0%)	82 (31.5%)	13 (20.3%)	0.018
**[Liver Function & Injury]**					
TP	69.10 (65.50,72.70)	69.60 (64.35,73.85)	66.20 (60.90,71.70)	61.80 (55.80,66.72)	< 0.001
Alb	42.80 (40.00,44.80)	40 (36,43)	33.75 (29.90,38.30)	32.10 (27.45,34.73)	< 0.001
GLB	26.30 (23.50,29.60)	28.80 (25.60,33.05)	31.25 (27.98,36.42)	29.70 (23.62,35.73)	< 0.001
Alb/GLB	1.60 (1.40,1.80)	1.40 (1.10,1.65)	1.10 (0.80,1.30)	1 (0,1)	< 0.001
ALT	28 (17,50)	30 (20,57)	25.50 (18.00,38.00)	54.50 (33.50,234.25)	< 0.001
AST	24 (19,36)	33 (23,59)	40 (27,61)	82 (59,199)	< 0.001
AST/ALT	0.90 (0.70,1.10)	1.10 (0.80,1.40)	1.50 (1.18,2.00)	1.25 (0.80,2.00)	< 0.001
GGT	31.50 (19.00,63.25)	46 (24,95)	42.50 (22.75,79.00)	72 (43,154)	< 0.001
ALP	74 (60,90)	92 (72,117)	112.50 (84.00,152.25)	140 (101,187)	< 0.001
TBil	11 (8,15)	16 (11,27)	22 (14,37)	140 (93,241)	< 0.001
DBil	3 (2,5)	5 (4,9)	9 (5,18)	100 (61,163)	< 0.001
IBil	8 (6,10)	10 (7,16)	12 (8,18)	49 (27,72)	< 0.001
ChE	8744 (7568,10178)	6520.50 (5168.25,7938.25)	3734 (2542,5217)	3000 (1829,4676)	< 0.001
TBA	4.70 (2.75,9.35)	16.50 (9.50,43.90)	49.20 (24.70,116.75)	212.30 (105.30,257.65)	< 0.001
GPDA	101 (83,123)	111.50 (90.00,139.25)	101 (82,128)	105 (80,129)	0.009
AFU	28 (23,33)	32 (28,38)	32 (25,40)	33 (28,46)	< 0.001
ADA	10 (8,14)	17 (13,23)	24 (19,30)	24 (19,29)	< 0.001
**[Coagulation]**					
PT	11.20 (10.70,12.00)	12.20 (11.40,13.30)	13.90 (12.60,15.95)	23.45 (18.40,30.92)	< 0.001
INR	0.98 (0.94,1.05)	1.07 (1.01,1.16)	1.23 (1.11,1.40)	2.00 (1.64,2.63)	< 0.001
APTT	31.70 (29.40,34.00)	33.60 (30.70,36.45)	35.20 (32.30,39.02)	40.60 (35.98,48.55)	< 0.001
TT	15.40 (14.60,16.40)	16.80 (15.60,18.10)	18.30 (16.90,19.90)	20.50 (19.25,22.75)	< 0.001
FIB	2.69 (2.35,3.12)	2.33 (2.00,2.76)	2.08 (1.63,2.64)	1.38 (1.09,1.88)	< 0.001
DD	80 (50,129)	98 (48,180)	264 (107,659)	912.50 (308.75,2600.75)	< 0.001
**[Inflammation & Hematology]**					
WBC	5.80 (4.80,7.10)	4.50 (3.55,5.60)	3.70 (2.77,5.00)	5.50 (4.00,6.88)	< 0.001
Neutrophil%	57.80 (51.40,64.30)	58.20 (51.75,64.55)	61.15 (52.83,69.67)	67.80 (60.08,78.03)	< 0.001
Lymphocyte%	31.65 (25.85,36.62)	29.70 (25.00,36.15)	24.60 (18.00,32.73)	18.80 (11.58,25.52)	< 0.001
Monocyte%	7.30 (6.07,8.72)	7.80 (6.70,9.80)	9.75 (7.40,11.80)	9.10 (7.15,11.80)	< 0.001
Eosinophil%	1.80 (1.10,2.70)	2 (1,3)	2 (1,3)	0.80 (0.30,1.92)	< 0.001
Basophils%	0.50 (0.30,0.70)	0.50 (0.30,0.70)	0.55 (0.30,0.90)	0.40 (0.10,0.60)	< 0.001
Neutrophil#	3.30 (2.60,4.20)	2.60 (1.85,3.30)	2.10 (1.50,3.12)	3.75 (2.48,5.10)	< 0.001
Lymphocyte#	1.70 (1.40,2.20)	1.40 (1.00,1.80)	0.90 (0.60,1.30)	0.90 (0.60,1.30)	< 0.001
Monocyte#	0.40 (0.30,0.50)	0.40 (0.30,0.50)	0.40 (0.20,0.60)	0.50 (0.30,0.70)	< 0.001
Eosinophil#	0.10 (0.06,0.16)	0.08 (0.05,0.15)	0.07 (0.04,0.15)	0.04 (0.01,0.10)	< 0.001
Basophils#	0.03 (0.02,0.04)	0.02 (0.01,0.03)	0.02 (0.01,0.04)	0.02 (0.01,0.03)	< 0.001
RBC	4.67 (4.30,5.05)	4.29 (3.81,4.72)	3.59 (3.10,4.14)	3.04 (2.41,4.00)	< 0.001
Hb	142 (131,153)	133 (117,147)	114 (95,131)	105 (88,128)	< 0.001
HCT	42.40 (38.90,45.00)	39.90 (35.10,43.15)	33.40 (28.40,38.15)	31.30 (24.75,36.90)	< 0.001
MCV	90.70 (88.10,93.33)	92.20 (89.05,95.55)	93.15 (87.38,99.90)	96.60 (90.50,103.35)	< 0.001
MCH	30.70 (29.70,31.60)	31.40 (30.05,32.50)	31.70 (28.60,34.30)	33.15 (31.27,35.15)	< 0.001
MCHC	338 (331,344)	338 (328,345)	338 (326,347)	345 (336,352)	< 0.001
RDW	12.50 (12.10,13.00)	13 (12,14)	14.60 (13.30,16.80)	16.15 (14.55,17.77)	< 0.001
PLT	198 (162,239)	115 (88,156)	76.50 (50.75,117.00)	68 (42,102)	< 0.001
PCT	0.22 (0.18,0.25)	0.13 (0.10,0.17)	0.09 (0.06,0.13)	0.08 (0.04,0.12)	< 0.001
MPV	10.60 (10.00,11.40)	11.20 (10.40,12.15)	11.10 (10.10,12.20)	10.70 (9.80,12.00)	< 0.001
PDW	13.30 (11.70,15.90)	14.20 (12.40,16.30)	15.30 (12.65,16.40)	15.80 (14.60,16.30)	< 0.001
CRP	5 (5,5)	5 (5,5)	5 (5,6)	10.80 (6.67,25.55)	< 0.001
hs-CRP	1.40 (0.50,3.10)	0.90 (0.20,3.15)	2.20 (0.60,5.00)	5 (5,5)	< 0.001
**[Composite Inflammatory Indices]**					
SII	370.28 (271.58,505.28)	224.89 (148.06,319.75)	198 (104,330)	305.27 (158.96,460.56)	< 0.001
NLR	1.81 (1.42,2.48)	1.94 (1.44,2.56)	2.50 (1.58,4.00)	3.64 (2.41,7.00)	< 0.001
PLR	109.45 (87.64,139.53)	87.37 (64.00,120.27)	90 (61,128)	71.80 (50.00,103.41)	< 0.001
NPAR	135.66 (121.03,152.59)	144.61 (127.60,163.10)	181.31 (145.68,213.32)	213.20 (184.33,254.80)	< 0.001
**[Metabolic & Renal Function]**					
Urea	4.80 (4.05,5.80)	5.10 (4.20,6.04)	5.17 (4.10,6.71)	4.77 (3.68,8.52)	0.019
Cr	66 (56,77)	66 (56,73)	67 (55,79)	68 (55,84)	0.582
UA	339 (279,409)	304 (255,366)	323 (255,414)	216 (141,329)	< 0.001
GFR	103.07 (94.20,111.09)	101.33 (88.72,109.82)	99.18 (84.83,111.67)	101.83 (77.38,114.93)	0.065
TC	4.67 (3.94,5.32)	4.20 (3.64,4.79)	3.54 (2.78,4.44)	2.56 (1.96,3.27)	< 0.001
TG	1.56 (1.06,2.49)	1.11 (0.79,1.54)	0.94 (0.68,1.42)	1.03 (0.77,1.50)	< 0.001
HDL	1.09 (0.91,1.28)	1.13 (0.92,1.34)	0.94 (0.71,1.18)	0.38 (0.19,0.55)	< 0.001
LDL	2.66 (2.06,3.28)	2.27 (1.81,2.80)	1.70 (1.23,2.35)	1.48 (1.04,2.10)	< 0.001
apoA1	1.12 (0.98,1.28)	1.12 (0.95,1.37)	0.94 (0.77,1.17)	0.44 (0.32,0.63)	< 0.001
apoB	0.89 (0.70,1.07)	0.80 (0.63,0.93)	0.64 (0.45,0.82)	0.66 (0.45,0.93)	< 0.001
CK	82 (62,112)	84 (59,110)	83.50 (62.25,130.00)	74 (43,134)	0.395
LDH	186 (166,213)	187 (165,216)	211 (182,258)	237 (205,307)	< 0.001
HBDH	138 (121,157)	142.50 (123.00,166.25)	169.50 (143.00,207.75)	184 (153,220)	< 0.001
**[Electrolytes]**					
K	3.89 (3.69,4.12)	3.88 (3.60,4.06)	3.81 (3.58,4.08)	3.81 (3.52,4.06)	0.026
Na	142 (140,143)	142 (140,143)	140 (138,142)	136.50 (134.00,139.00)	< 0.001
Cl	105 (104,107)	106 (104,108)	106 (103,108)	103.50 (100.75,106.00)	< 0.001
Ca	2.27 (2.19,2.34)	2.24 (2.15,2.31)	2.18 (2.08,2.28)	2.11 (2.04,2.27)	< 0.001
P	1.11 (1.00,1.23)	1.07 (0.95,1.19)	1.04 (0.92,1.17)	0.94 (0.75,1.23)	< 0.001
**[Thyroid Function]**					
T3	1.52 (1.35,1.73)	1.44 (1.23,1.67)	1.15 (0.91,1.43)	0.73 (0.57,0.89)	< 0.001
T4	104.35 (91.30,117.47)	104.80 (88.05,119.90)	88.10 (74.50,103.40)	71.65 (47.75,106.15)	< 0.001
FT3	4.55 (4.12,5.00)	4.20 (3.74,4.63)	3.50 (3.03,4.10)	2.83 (2.09,3.10)	< 0.001
FT4	14.95 (12.47,17.18)	13.84 (11.35,15.96)	13.66 (11.42,15.56)	16.15 (12.26,18.31)	< 0.001
TSH	1.82 (1.30,2.70)	1.91 (1.46,2.83)	2.04 (1.35,3.10)	0.98 (0.44,2.54)	< 0.001
**[Urinalysis]**					
USG	1.02 (1.01,1.03)	1.02 (1.01,1.02)	1.02 (1.01,1.02)	1.02 (1.01,1.02)	< 0.001
Urine pH	6 (5,6)	6.50 (6.00,7.00)	6.50 (6.00,7.00)	6.50 (6.00,7.00)	< 0.001

Footnote: Data are presented as median (interquartile range, IQR) for continuous variables and n (%) for categorical variables. P values were calculated using the Kruskal-Wallis test for continuous variables and the chi-square test or Fisher’s exact test for categorical variables, as appropriate

**Table 2:** Gut microbiota abundance and derived functional ratios

Variable	CLD (*n* = 464)	CC (*n* = 159)	DC (*n* = 260)	ACLF (*n* = 64)	*P*
**[Raw Abundance]**					
*Enterococcus*	18,900 (2587–107500)	17,000 (1415–145000)	16,950 (2365–129000)	157,000 (13975–666500)	< 0.001
*Lactobacillus*	135,500 (10250–3075000)	147,000 (7380–5850000)	88,250 (13025–2480000)	545,000 (60850–3757500)	0.151
*Clostridium leptum*	306,500 (19500–2995000)	548,000 (19350–3375000)	642,500 (18525–3242500)	104,900 (5530–891000)	0.021
*Clostridium butyricum*	1,515,000 (64175–27925000)	284,000 (14550–8935000)	106,000 (6037–2562500)	178,000 (9432–2897500)	< 0.001
*Eubacterium rectale*	157,000 (6312–2475000)	88,900 (7545–874500)	4660 (444–144250)	3260 (163–24100)	< 0.001
*Faecalibacterium prausnitzii*	1,480,000 (196000–10050000)	1,240,000 (146500–9625000)	968,000 (76875–5612500)	263,000 (3785–4222500)	< 0.001
*Bacteroides*	7.26 (5.64–8.12)	7.29 (6.57–8.11)	7.21 (6.30–7.74)	6.77 (5.42–7.54)	0.054
*Enterobacterium*	627,500 (65300–5542500)	416,000 (35200–4110000)	226,000 (31325–1822500)	551,500 (16000–6865000)	0.008
*Bifidobacterium*	77,100 (6885–1797500)	66,000 (5325–860500)	41,650 (5582–360750)	168,500 (10647–1260000)	0.046
*Atopobium cluster*	5.48 (4.32–6.78)	5.08 (4.11–6.44)	4.55 (3.85–5.61)	5.04 (4.00–5.94)	< 0.001
**[Derived Ratios]**					
E/Er Ratio	0.08 (0.00–2.98)	0.08 (0.01–3.40)	3.60 (0.07–43.67)	45.67 (3.54–1767.81)	< 0.001
Pro/Anti-inflammatory Ratio	0.05 (0.01–0.35)	0.03 (0.01–0.16)	0.05 (0.01–0.38)	0.25 (0.02–2.35)	< 0.001
Butyrate-Pro Ratio	11.22 (1.89–124.89)	20.14 (2.43–133.32)	10.66 (1.09–68.81)	2.69 (0.06–16.58)	< 0.001
Butyrate-Total Ratio	0.21 (0.08–0.43)	0.16 (0.05–0.33)	0.16 (0.05–0.32)	0.10 (0.01–0.37)	< 0.001
Specific-Butyrate-Total Ratio	0.05 (0.02–0.13)	0.04 (0.01–0.11)	0.04 (0.01–0.11)	0.02 (0.00–0.12)	0.002
Specific-Butyrate-Pro Ratio	3.66 (0.29–46.64)	5.41 (0.38–46.82)	3.02 (0.11–25.73)	0.65 (0.00–6.17)	< 0.001

Footnote: Raw abundances are log10-transformed; ratios are presented as raw values

#### Hepatic function and synthetic capacity

Indices reflecting hepatic synthetic function demonstrated a progressive decline from CLD (G1) to ACLF (G4). Levels of Albumin (Alb) and Cholinesterase (ChE) showed a continuous decrease. The median Alb dropped significantly from 42.8 g/L (IQR: 40, 44.8) in CLD to 32.1 g/L (IQR: 27.35, 34.775) in ACLF. Similarly, median ChE plummeted from 8744 U/L in CLD to 3000 U/L in ACLF, signifying severe exhaustion of hepatic synthetic reserve.

Conversely, Total Bilirubin (TBil), Direct Bilirubin (DBil), and Total Bile Acid (TBA) all showed a step-wise increase, reflecting worsening cholestasis. Median TBil rose from 11 µmol/L in CLD to 22 µmol/L in DC, peaking at 140 µmol/L in the ACLF group. Markers of hepatocyte injury, Alanine Aminotransferase (ALT) and Aspartate Aminotransferase (AST), were highest in the ACLF (G4) group. Furthermore, the AST/ALT ratio increased with disease progression, rising from 0.9 in CLD to 1.5 in DC, suggesting escalating degrees of liver fibrosis and cirrhosis.

#### Inflammation and immune response

Complex changes in inflammation and immune cell counts were observed during disease progression. C-Reactive Protein (CRP) remained relatively low during CLD (G1, 5 mg/L) through DC (G3, 5 mg/L), but surged significantly in the ACLF (G4) group to 10.8 mg/L (IQR: 6.425, 26.25), highlighting systemic inflammatory burst as a hallmark of ACLF.

White Blood Cell (WBC) count was lowest in the DC group (3.7 × 10^9^/L) but rose again in the ACLF group (5.5 × 10^9^/L). The Neutrophil percentage (Neutrophil%) continuously increased from 57.8% in CLD to 67.8% in ACLF, while the Lymphocyte percentage (Lymphocyte%) steadily decreased. This pattern translates to an elevated Neutrophil-to-Lymphocyte Ratio (NLR), consistent with the immune dysfunction and hyper-inflammatory state observed in DC and ACLF. Platelet Count (PLT) significantly decreased with progression, reaching a median of 68 × 10^9^/L in the ACLF group, reflecting potential worsening portal hypertension and hypersplenism.

#### Metabolic and nutritional status

Hepatic dysfunction, as the primary metabolic organ, was reflected in profound changes in lipid and nutritional indices. Levels of Total Cholesterol (TC), Low-Density Lipoprotein (LDL), High-Density Lipoprotein (HDL), and Apolipoprotein A1 (apoA1) all demonstrated progressive decline from CLD to ACLF. Specifically, TC decreased from 4.67 mmol/L in CLD to 2.555 mmol/L in ACLF, and apoA1 dropped from 1.12 g/L to 0.44 g/L. This state of hypolipidemia is a typical sign of severely impaired hepatocyte lipoprotein synthesis and hepatic failure.

Uric Acid (UA) levels were higher in the CLD (339 µmol/L) and CC/DC (304–323 µmol/L) groups but significantly decreased in the ACLF group (216 µmol/L), possibly due to poor nutritional status and inadequate synthesis. Thyroid Hormone levels (T3, FT3) were notably reduced in the ACLF group (0.735 nmol/L, 2.835 pmol/L), consistent with the “euthyroid sick syndrome” (or low T3 syndrome) associated with severe liver disease and poor prognosis.

#### Electrolyte and renal function

While Creatinine (Cr) and estimated Glomerular Filtration Rate (eGFR) showed no significant clinical differences across groups (*P* > 0.05), electrolyte disturbances were more pronounced in the ACLF group. Serum Sodium (Na) levels decreased with disease severity, dropping from 142 mmol/L in CLD to 136.5 mmol/L in ACLF, reflecting dilutional hyponatremia due to increased anti-diuretic hormone secretion in advanced liver disease. Serum Calcium (Ca) and Phosphorus (P) also trended downward with disease progression.

#### Coagulation dysfunction

Coagulopathy represents another critical feature of hepatic failure. Prothrombin Time (PT), International Normalized Ratio (INR), Activated Partial Thromboplastin Time (APTT), and Thrombin Time (TT) all progressively prolonged from CLD to ACLF. INR acutely worsened from a median of 0.98 (CLD) to 2.005 (ACLF). Fibrinogen (FIB) continuously declined, dropping from 2.69 g/L in CLD to 1.38 g/L in ACLF, indicating severe impairment of hepatic clotting factor synthesis. D-dimer (DD) levels sharply increased, soaring from 80 µg/L in CLD to 912.5 µg/L in ACLF, strongly suggesting a propensity for Disseminated Intravascular Coagulation (DIC) or secondary hyperfibrinolysis in ACLF patients. The sustained decline in PLT count from 198 × 10^9^/L (CLD) to 68 × 10^9^/L (ACLF) aligns with the typical presentation of hypersplenism in cirrhosis.

The baseline data of this cohort clearly delineate a multi-system, progressive pattern of functional derangement from CLD to ACLF. ACLF patients exhibited the most severe hepatocyte injury, synthetic failure, intense systemic inflammatory response, and significant metabolic and coagulation abnormalities. This provides a robust clinical background for the subsequent investigation into the role of the gut microbiota in these pathophysiological processes.

### Correlation analysis between gut microbiota and clinical indices

#### Extensive association of key gut microbiota with systemic pathophysiology of liver disease progression

This study performed a comprehensive correlation analysis between the relative abundance of the specified dominant gut microbiota and a comprehensive panel of clinical laboratory parameters, providing critical insights into the interplay between gut dysbiosis and the systemic pathophysiology of progressive liver disease.

The resulting correlation matrix was visualized using a hierarchical clustering heatmap (Fig. 2). As shown in Fig. 2A, the overall analysis (encompassing the entire cohort from CLD to ACLF) detected a total of 93 robust and significant associations (after FDR correction), comprising 49 positive correlations and 44 negative correlations. This substantial number of statistically significant links underscores the broad and powerful systemic impact of gut microbiota dysbiosis on the host’s overall pathophysiology throughout the course of chronic liver disease progression. This pervasive association pattern strongly supports the core concept of the “Gut-Liver Axis”, suggesting that alterations in the gut microbiome are closely intertwined with systemic inflammation, hepatocyte dysfunction, and coagulation abnormalities throughout the course of chronic liver disease progression.

#### Universal core microbial markers and their role in disease severity

The analysis highlighted that the relative abundance of 10 dominant gut microbiota exhibited significant correlations with multiple clinical markers of hepatic function, inflammation, and coagulation, suggesting their potential utility as microbial biomarkers across the entire disease spectrum.

##### *A. Enterococcus* overgrowth and its association with hepatic failure and systemic inflammation

*Enterococcus* demonstrated the strongest and most pervasive associations in the overall cohort. The abundance of this genus was significantly negatively correlated with the hepatic synthetic index, Albumin (Alb) (*r* = -0.355, Bonferroni corrected *P*-value: 1.95 × 10^− 6^), and significantly positively correlated with markers of hepatocyte injury and cholestasis, Total Bilirubin (TBil) (*r* = 0.231, Bonferroni corrected *P*-value: 3.70 × 10^− 5^) and Direct Bilirubin (DBil) (*r* = 0.370, Bonferroni corrected *P*-value: 3.42 × 10^− 11^). The pattern of “low albumin, high bilirubin” is the classic clinical signature of severe hepatic insufficiency and failure. The highly significant association between *Enterococcus* and this pattern suggests that *Enterococcus* proliferation may be a core microbial characteristic of worsening liver function, irrespective of the specific disease stage. Furthermore, *Enterococcus* showed a significant positive correlation with high-sensitivity C-Reactive Protein (hs-CRP) (*r* = 0.203, Bonferroni corrected *P*-value: 1.04 × 10^− 10^), tightly linking it to the systemic inflammatory response observed in chronic liver disease progression.

##### B. Short-chain fatty acid (SCFA)-producing bacteria and protective effects

*Eubacterium rectale*, a primary butyrate producer, showed a significant negative correlation with markers of hepatic failure (e.g., TBil) in the overall cohort. This aligns with the proposed mechanism where butyrate reduces the inflammatory burden on the liver by strengthening intestinal tight junctions and reducing bacterial translocation.

Microbiota responsible for producing SCFAs, such as *Bifidobacterium*, *Eubacterium rectale*, and *Faecalibacterium prausnitzii*, are generally considered key custodians of gut health and barrier function.

This inverse association pattern suggests that the relative enrichment of these microbiota may exert a potential protective effect on the maintenance of hepatic detoxification and synthetic function, which is consistent with the known anti-inflammatory and barrier-enhancing effects of SCFAs on the gut and liver.

#### Interpretation of disease stage-specific correlations

Analysis of stage-specific correlation results revealed the dynamic nature of the gut microbiota-clinical parameter relationship as the disease advances, as visualized in the stage-specific heatmaps (Fig. 2B-E):

##### Early stage (CLD, Stage 1)

This stage displayed the highest number of significant positive correlations (31 positive, 4 negative) after FDR correction. This suggests that in the initial stages of the disease, the increase in abundance of certain microbes co-occurs with the host’s initial inflammatory response or compensatory mechanisms. For instance, the significant positive correlation of *Enterococcus* with TBil, DBil, and hs-CRP was already established (all Bonferroni corrected *P*-values signficant), suggesting that dysbiosis begins to exert its pathogenic effect even at the CLD stage.

##### Middle stage (CC and DC, stage 2 and 3)

In the CC and DC stages, the number of significant correlations decreased, suggesting a transitional and unstable state of the gut-liver axis interaction.

##### Late stage (ACLF, Stage 4)

In the ACLF stage, both Bonferroni and FDR corrections resulted in zero significant associations. Although underlying trends still existed (e.g., *Enterococcus* was negatively correlated with Alb), statistical significance was lost. This phenomenon is likely attributable to two factors: (a) In extremely late-stage disease, systemic inflammation and liver failure have reached a state of saturation, where the clinical phenotype is primarily driven by the severity of organ failure, and subtle differences in microbial abundance are no longer the primary factor driving linear correlations; (b) The extremely severe dysbiosis leads to a sharp reduction in microbial diversity, resulting in a highly homogenized community structure.

### Overall dynamic evolution of gut microbiota during liver disease progression

**Fig. 3:**
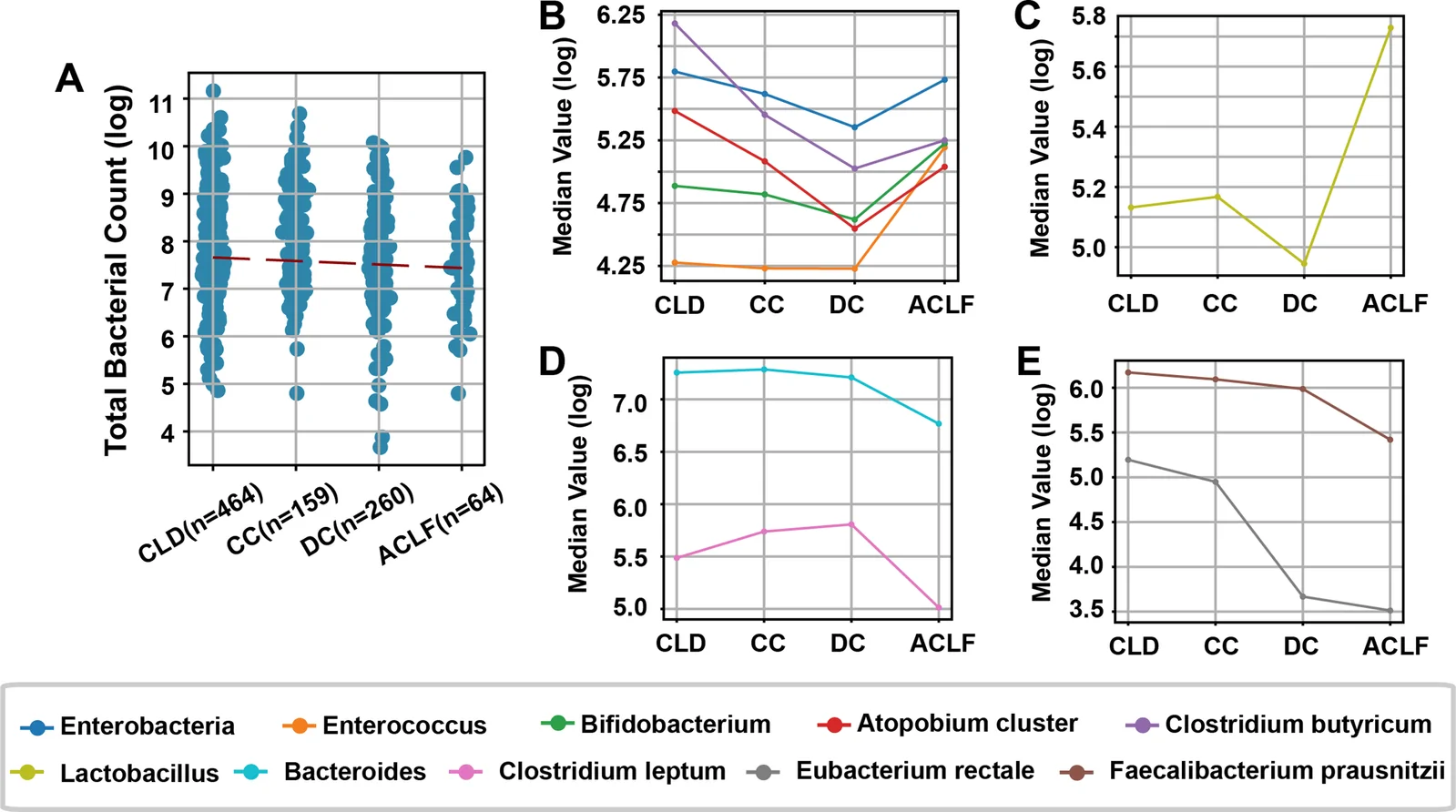
Evolutionary patterns of gut microbiota quantity and taxon abundance across different stages of chronic liver disease. (**A**) Total Bacterial Load: Scatter plots representing the total bacterial counts (log-transformed) across the four disease stages. The red dashed line connects the median values of each group, indicating a progressive decline in overall microbial density as the disease advances. (**B**) " Decline-then-Rise " Pattern: Trends for *Enterococcus*,* Enterobacteria*,* Bifidobacterium*,* Atopobium cluster*, and *Clostridium butyricum*. These microbiota exhibit a characteristic decrease from the CLD stage to DC, followed by a significant rebound during the ACLF stage. (**C**) “Fluctuating-Rise” Pattern: The abundance of *Lactobacillus* shows minor fluctuations during the early stages of disease but increases sharply upon the onset of ACLF. (**D**) “Rise-then-Decline” Pattern: Evolutionary trends for *Bacteroides* and *Clostridium leptum*, characterized by a relative increase during the transition from CLD to DC, followed by a precipitous decline in the ACLF stage. (**E**) “Progressive Depletion” Pattern: Key butyrate-producing bacteria, *Eubacterium rectale* and *Faecalibacterium prausnitzii*, exhibit a continuous and significant reduction throughout the entire spectrum of liver disease progression

To systematically assess the role of the gut microbiota in the dynamic progression of chronic liver disease, we conducted a longitudinal analysis of the abundance changes for the 10 dominant gut microbiota. Based on their trajectory of change across the different disease stages (from CLD to ACLF), these bacteria were clearly categorized into four distinct dynamic patterns (Fig. 3). Furthermore, based on the immunological function of the microbiota, we calculated the ratio of pro-inflammatory to anti-inflammatory bacteria and analyzed the relative abundance of butyrate-producing bacteria to elucidate their functional significance.

#### Four dynamic evolutionary patterns of microbial composition

##### “Decline-then-rise” pattern

This pattern encompassed five microbiota: *Enterococcus*,* Enterobacterium*,* Bifidobacterium*,* Atopobium cluster*, and *Clostridium butyricum*. These bacteria showed a slight decline in abundance during the early stages of the disease (CLD to CC), but exhibited a significant rebound as the disease entered the late stages (DC to ACLF). Notably, the acute proliferation of *Enterococcus* and *Enterobacterium*—both typical opportunistic pathogens—in the ACLF stage strongly suggests a close link with severe impairment of intestinal barrier function, increased risk of bacterial translocation, and exacerbated systemic inflammation.

##### “Fluctuating-rise” pattern

*Lactobacillus* was the sole representative of this pattern. Its abundance exhibited a fluctuating but overall rising trend throughout the disease course, peaking significantly in the ACLF stage. This unique growth trajectory may reflect an adaptive advantage of this genus in the disordered gut microenvironment or suggest a specialized role for certain strains in advanced liver disease.

##### “Rise-then-decline” pattern

This pattern included *Bacteroides* and *Clostridium leptum*. Their abundance peaked during the CC stage and subsequently began to decline upon entering the DC. This trajectory likely reflects a compensatory microbial reshaping in the early phase of the disease, with the subsequent decline signaling the start of further gut environmental deterioration and the collapse of the microbial ecosystem.

##### “Progressive depletion” pattern

This pattern included the most critical butyrate-producing bacteria: *Eubacterium rectale* and *Faecalibacterium prausnitzii*. The abundance of these two bacteria showed a stable and continuous decline starting from the early stage (CLD) and reaching its nadir in the ACLF stage. Given their core role in maintaining intestinal barrier integrity, producing the anti-inflammatory substance butyrate, and regulating immune homeostasis, their depletion is one of the most characteristic changes in liver disease-associated gut dysbiosis and holds significant value for early warning.

#### Disruption of the pro-inflammatory and anti-inflammatory microbial balance

**Fig. 4:**
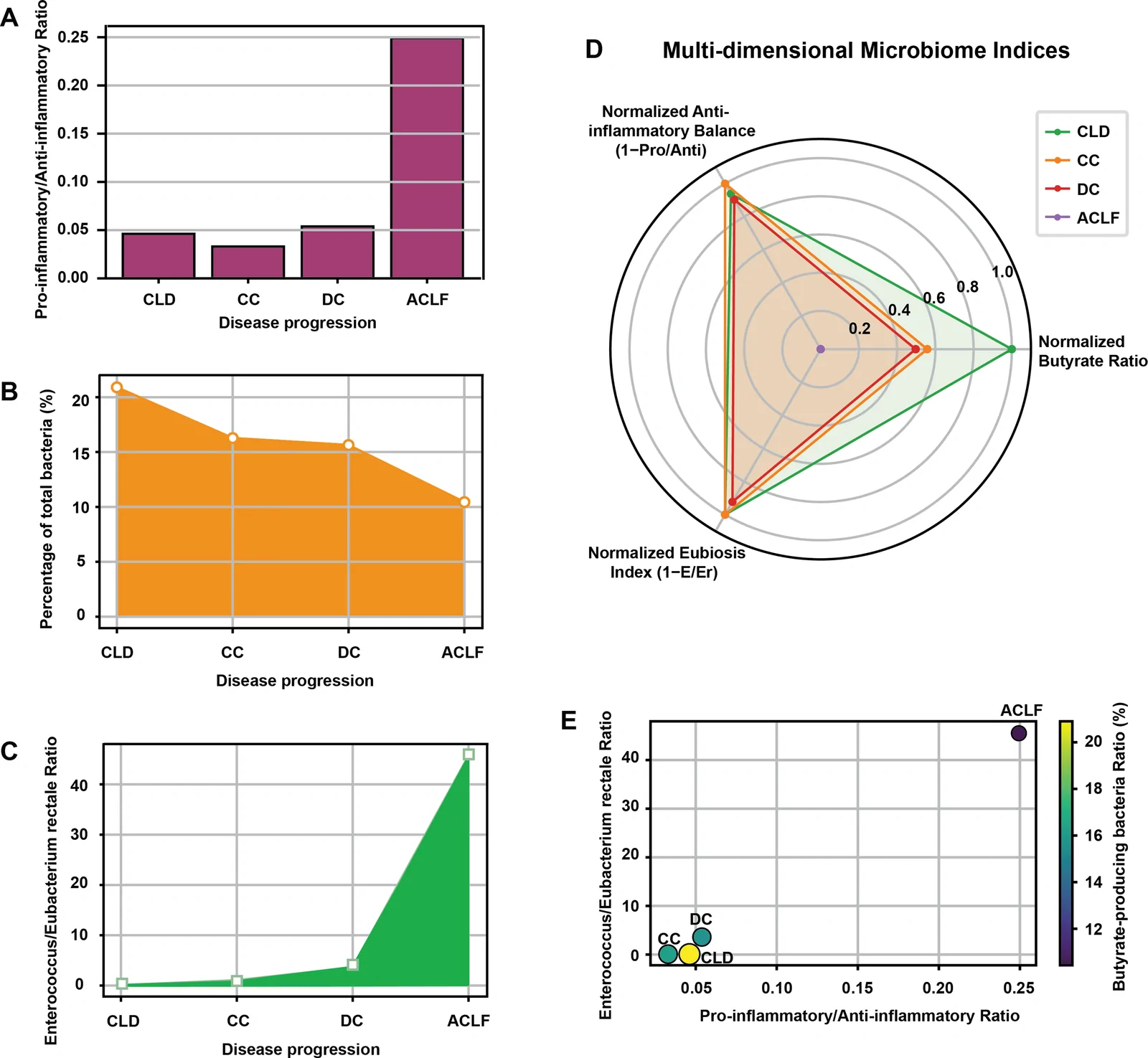
Functional and ratio-based distributional characteristics of the gut microbiota across stages of chronic liver disease progression. (**A**) Pro-inflammatory/anti-inflammatory ratio: A bar chart illustrating the shifting balance between pro-inflammatory and anti-inflammatory microbiota across the four disease stages (CLD, CC, DC, and ACLF). Values represent the derived indices reflecting systemic microbial inflammatory potential. (**B**) Percentage of Butyrate-producing Bacteria: An area chart showing the progressive depletion of butyrate-producing microbiota as a percentage of the total microbial community throughout the disease spectrum. **(C**) *Enterococcus*/*Eubacterium rectale* E/Er Ratio: An area chart depicting the dynamic changes in the E/Er Ratio, highlighting the sharp escalation in the ratio during the transition to the ACLF stage. (**D**) Multi-dimensional Microbial Indices: A radar chart integrating three normalized metrics: Normalized Butyrate Ratio, Normalized Anti-inflammatory Balance (1 – Pro-inflammatory/Anti-inflammatory Ratio), and Normalized Eubiosis Index (1 – E/Er Ratio). Each index was independently normalized to a 0–1 scale via Min–Max scaling across the four groups, with 1.0 representing the most beneficial state. The progressive contraction of the radar area from CLD to ACLF reflects the coordinated loss of beneficial microbial functions with advancing liver disease. (**E**) Bivariate Relationship Analysis: A scatter plot illustrating the correlation between the Pro-inflammatory/Anti-inflammatory Ratio (x-axis) and the E/Er Ratio (y-axis). Each data point represents an individual patient, color-coded by disease stage. The bubble size is proportional to the total percentage of butyrate-producing bacteria (right-side vertical color bar), emphasizing the synergistic collapse of microbial homeostasis in advanced liver failure. Sample sizes: CLD, *n* = 464; CC, *n* = 159; DC, *n* = 260; ACLF, *n* = 64

To quantify the overall immunomodulatory inclination of the gut microbiota, we calculated the ratio of pro-inflammatory microbiota (*Enterococcus*, *Enterobacterium*) to anti-inflammatory microbiota (*Lactobacillus*,* Bifidobacterium*,* Clostridium butyricum*,* Clostridium leptum*,* Eubacterium rectale*,* Faecalibacterium prausnitzii*). The results demonstrated a sharp increase in this pro-inflammatory/anti-inflammatory ratio during liver disease progression, accumulating a 5.4-fold increase from the CLD to the ACLF stage (Fig. 4A). The sharp surge in this ratio vividly indicates that as liver disease worsens, the overall balance of the gut microenvironment irreversibly shifts from one that maintains immune homeostasis to one that drives a systemic inflammatory state.

#### Functional failure of butyrate-producing bacteria

We further analyzed the relative proportion of butyrate-producing bacteria (including *Eubacterium rectale*, *Faecalibacterium prausnitzii*, *Clostridium leptum*, and *Clostridium butyricum*) within the total bacterial population. As shown in Fig. 4B, the proportion of butyrate-producers significantly and continuously decreased with disease progression. This not only corroborates the depletion of the species in the “Sustained-Decline” pattern but also reveals the functional loss of ecosystem services in the gut during the liver disease process. The reduction in butyrate is directly linked to insufficient energy supply for intestinal epithelial cells and downregulation of tight junction protein expression, ultimately leading to gut barrier dysfunction, which provides a pathway for sustained activation of endotoxemia and intrahepatic inflammation.

#### The enterococcus/eubacterium rectale ratio

The dynamic change in the E/Er Ratio (Fig. 4C) further confirmed the functional microbial dysbiosis. The ratio, representing the balance between the potential opportunistic pathogen (*Enterococcus*) and the core anti-inflammatory symbiont (*Eubacterium rectale*), showed an explosive increase from CLD (G1) to ACLF (G4) (*P* < 0.001). This dramatic shift marks the replacement of the core anti-inflammatory community by potentially pathogenic or pro-inflammatory bacteria, serving as a classic microbiological signature of liver disease decompensation.

#### Comprehensive assessment of microbiome-derived indices

A radar chart (Fig. 4D) was constructed by integrating three normalized microbiome‑derived metrics: Normalized Butyrate Ratio, Normalized Anti‑inflammatory Balance, and Normalized Eubiosis Index. Each index was independently scaled to a 0–1 range via Min–Max normalization, with 1.0 representing the most beneficial state observed across the four disease stages.

In the CLD group, all three indices were at or near their maximum, producing the largest radar chart area, consistent with relatively preserved gut homeostasis. As the disease progressed, all three indices declined progressively. In the ACLF group, all three indices reached 0.0, reflecting the simultaneous collapse of butyrate‑producing capacity, anti‑inflammatory balance, and eubiotic community structure. The radar chart area thus contracted from a broad polygon in CLD to a single point at the origin in ACLF, graphically illustrating the systemic failure of the gut ecosystem in end‑stage liver disease.

### Construction of random forest models for predicting disease progression

Before constructing the predictive models, we first evaluated the relationship between microbial features and disease severity. As illustrated by the dynamic evolutionary trends in Figs. 3 and 4, the abundance of various microbiota—particularly the E/Er Ratio—exhibited stage-specific fluctuations rather than simple monotonic or linear changes. Given these inherent non-linear characteristics and the complex interactions within the gut-liver axis, we selected the Random Forest (RF) algorithm for modeling. RF is a robust machine learning approach capable of capturing high-dimensional, non-linear dependencies without requiring prior assumptions about data distribution.

To systematically evaluate the predictive value of clinical and microbiological characteristics across different stages of liver disease, we developed Random Forest (RF) models to predict three critical transitions: CLD → CC, CC → DC, and DC → ACLF. These models integrated key clinical parameters (PT, Alb, and TBil) with various microbiome-derived indices (e.g., butyrate-related ratios, E/Er Ratio). Feature selection was optimized via cross-validation to maximize discriminatory efficacy.

To quantify the incremental value of microbiome-derived features, we compared three Random Forest model variants for each transition: Clinical-only (TBil, Alb, PT), Microbiome-only (the six ratio features), and Combined (clinical + microbiome). Model performance was assessed using AUC, Accuracy, Precision, Recall, and F1-score on the held-out test set (Supplementary Table S4).

Across all three transitions, the Combined model consistently outperformed both individual models. For the transition from CLD to CC, the Combined model achieved an AUC of 0.735 compared to 0.628 for Clinical-only and 0.511 for Microbiome-only, with corresponding improvements in Precision (0.500 vs. 0.353) and F1-score (0.314 vs. 0.293). For CC to DC, the Combined model achieved an AUC of 0.706 (vs. 0.683 Clinical-only), with Recall reaching 0.795 and F1-score of 0.747. In the DC to ACLF transition, where clinical markers already provided near-perfect discrimination (AUC 0.954), the Combined model further increased the AUC to 0.958, and Precision improved from 0.739 to 0.789, indicating a reduction in false positives. Notably, the Microbiome-only models performed poorly across all tasks (AUC range: 0.511–0.679), underscoring that clinical parameters remain essential and that microbiome features serve a complementary rather than standalone predictive role.

#### Progression from CLD to CC (Transition Group A)

Feature importance analysis revealed that Prothrombin Time (PT), Albumin (Alb), and Total Bilirubin (TBil) played dominant roles in the prediction. Among the microbiome-derived indices, the Butyrate-to-Pro-inflammatory ratio and the Specific-Butyrate-to-Total ratio also provided substantial contributions. The RF model achieved an Area Under the Receiver Operating Characteristic Curve (AUC) of 0.744 (Fig. 4A), indicating moderate discriminatory power for this early transition. Cross-validation determined that the model reached its peak performance when five microbiome features were included, yielding a cross-validation AUC of 0.740.

#### Progression from CC to DC (Transition Group B)

**Fig. 5:**
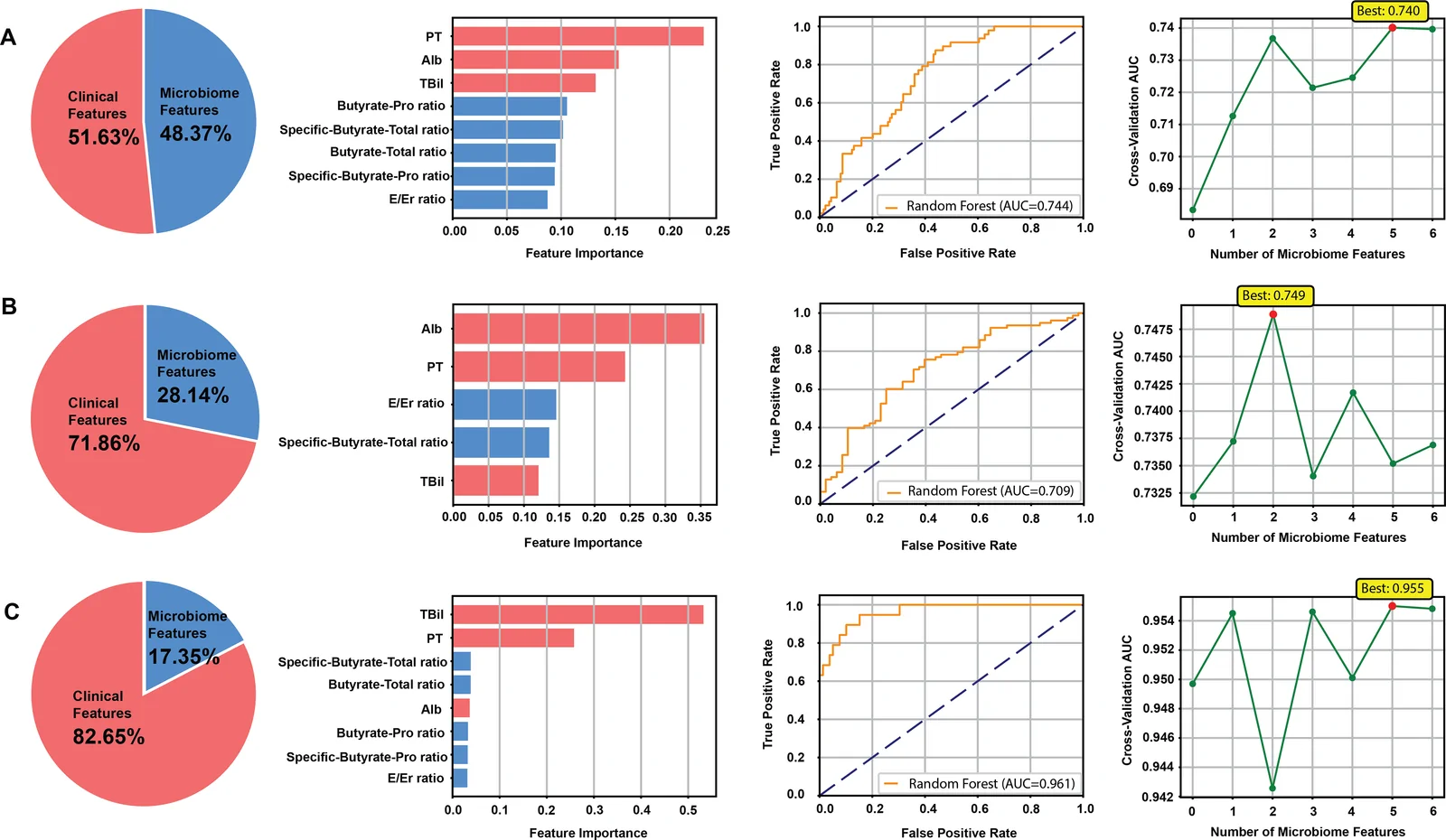
Predictive performance of random forest models across stages of chronic liver disease progression.This figure illustrates the integrated performance of Random Forest models in predicting three critical clinical transitions (Panels A, B, and C), combining traditional clinical parameters (red) with microbiome-derived features (blue). (**A**) Prediction of Progression from CLD to CC: Pie Chart: Illustrates the relative contribution of clinical features (51.63%) versus microbiome features (48.37%) to the overall model.Bar Chart: Displays feature importance scores, highlighting the significant role of parameters such as Prothrombin Time (PT), Albumin (Alb), and butyrate-related microbial ratios. ROC Curve: Demonstrates the model’s discriminatory power with an AUC of 0.744. On the held-out test set, the Combined model achieved a Precision of 0.500, Recall of 0.229, and F1-score of 0.314. Validation Plot: Shows the relationship between cross-validation AUC and the number of microbiome features, identifying an optimal “Best” AUC of 0.740. (**B**) Prediction of Progression from CC to DC: Pie Chart: Shows a dominant weight of clinical features (71.86%) compared to microbiome features (28.14%) during this transition. Bar Chart: Identifies Alb and PT as primary clinical predictors, while the *Enterococcus/Eubacterium* ratio emerges as a key microbial feature. ROC Curve: Demonstrates a model AUC of 0.709. On the held-out test set, the Combined model achieved a Precision of 0.705, Recall of 0.795, and F1-score of 0.747. Validation Plot: Displays the optimization of microbiome features to achieve a “Best” cross-validation AUC of 0.749. (**C**) Prediction of Progression from DC to ACLF: Pie Chart: Indicates that clinical features contribute the vast majority of predictive weight (82.65%), reflecting the “ceiling effect” of extreme biochemical abnormalities in end-stage failure. Bar Chart: Highlights the overwhelming importance of Total Bilirubin (TBil) and PT in predicting the transition to ACLF. ROC Curve: Demonstrates exceptional discriminatory power with an AUC of 0.961. On the held-out test set, the Combined model achieved a Precision of 0.789, Recall of 0.789, and F1-score of 0.789. Validation Plot: Identifies an optimal cross-validation AUC of 0.955. Precision, Recall, and F1‑score for each model are provided in Supplementary Table S4

In this stage, Alb and PT remained significant predictors; however, the contribution of the E/Er Ratio and the Specific-Butyrate-to-Total ratio increased markedly. The initial model achieved an overall AUC of 0.709 (Fig. 5B), showing slightly lower predictive capacity than the previous stage. However, cross-validation results demonstrated that the model performance was optimized when only two core microbiome features were included, with the AUC rising to 0.749, suggesting improved robustness after targeted feature selection.

#### Progression from DC to ACLF (Transition Group C)

The predictive performance of the model improved significantly for the transition to ACLF, with an AUC of 0.961 (Fig. 5C), demonstrating exceptionally strong discriminatory capability. According to the feature importance ranking, TBil and PT were identified as the most critical factors. Microbial features, specifically the Specific-Butyrate-to-Total ratio and the Butyrate-to-Total ratio, also carried high weights in the model. The optimized cross-validation AUC reached 0.955 with the inclusion of five key microbiome features.

#### Definition of microbiome-derived ratios

To quantify the functional shifts in the gut ecosystem, the following four microbiome-derived indices were calculated based on the targeted microbiota:

##### Butyrate-pro ratio

Represents the ecological balance between all detected butyrate-producing bacteria (*Eubacterium rectale*,* Faecalibacterium prausnitzii*,* Clostridium leptum*,* Clostridium butyricum*) and opportunistic pro-inflammatory pathogens (*Enterococcus and Enterobacterium*).

##### Specific-butyrate-total ratio

Reflects the relative abundance of the two most critical anti-inflammatory commensals (*Eubacterium rectale*, *Faecalibacterium prausnitzii*) within the total targeted 10-taxon microbial community.

##### Butyrate-total ratio

Indicates the collective proportion of all identified butyrate-producing species relative to the total targeted 10-taxon microbial community.

##### Specific-butyrate-pro ratio

Specifically measures the ratio of the primary butyrate-producers (*Eubacterium rectale*, *Faecalibacterium prausnitzii*) to the core pro-inflammatory microbiota (*Enterococcus and Enterobacterium*), serving as a refined indicator of intestinal homeostasis.

### Discriminatory efficacy of the E/Er ratio as a predictive biomarker

To further evaluate the predictive value of a specific microbiome-derived marker—the E/Er Ratio—at critical transition points of liver disease, we constructed predictive models to distinguish between CC and DC, and between DC and ACLF. The discriminatory efficacy of these models was assessed using ROC curve analysis.

**Fig. 6:**
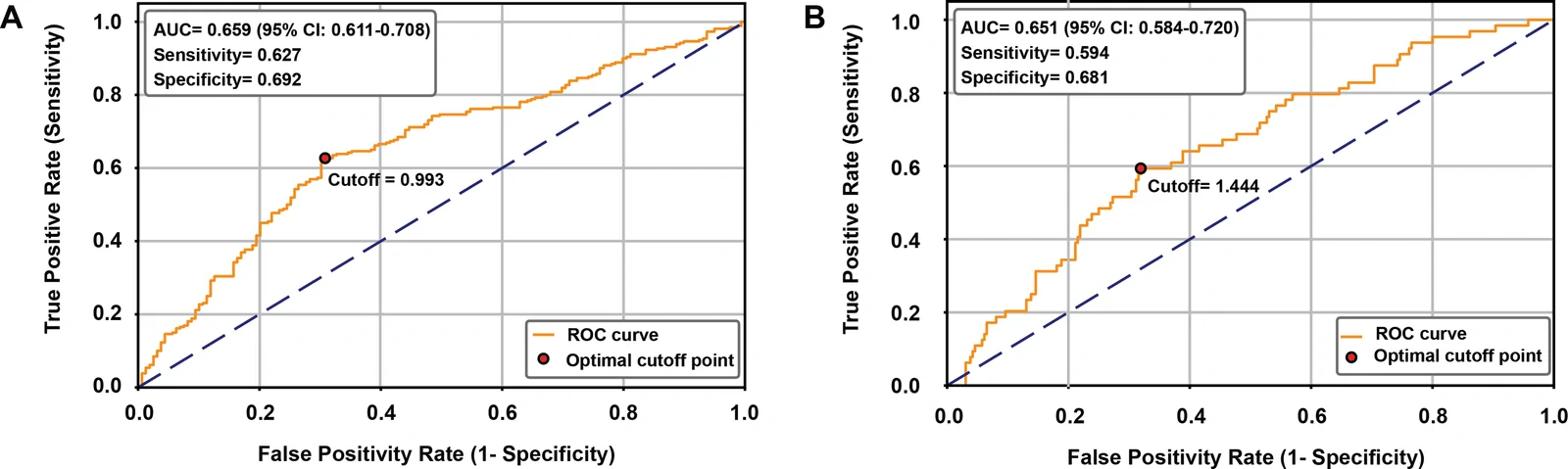
Diagnostic efficacy and optimal cutoff identification of the *Enterococcus/Eubacterium* rectale (E/Er) ratio.The Receiver Operating Characteristic (ROC) curves illustrate the discriminatory power of the E/Er Ratio in identifying disease progression across the chronic liver disease spectrum. (**A**) Identification of CC: The ROC curve demonstrates the efficacy of the E/Er Ratio in distinguishing CC from CLD. The model achieved an AUC of 0.659 (95% CI: 0.611–0.708), with a sensitivity of 0.627 and a specificity of 0.692. An optimal cutoff value of 0.993 was identified (red dot). (**B**) Identification of DC: The ROC curve evaluates the E/Er Ratio’s performance in identifying DC progression. The analysis yielded an AUC of 0.651 (95% CI: 0.584–0.720), with a sensitivity of 0.594 and a specificity of 0.681. An optimal cutoff value of 1.444 was determined (red dot)

#### Differentiation between CC and DC stages

The E/Er Ratio exhibited significant differences between CC patients (*n* = 159) and DC patients (*n* = 260). The median ratio in the CC group was 0.80 (IQR: 0.63–1.17), whereas it significantly increased to 1.19 (IQR: 0.79–1.66) in the DC group. ROC curve analysis revealed that the AUC for differentiating CC from DC was 0.659 (95% CI: 0.611–0.708) (Fig. 6A), which was statistically significant (*P* < 0.05). At the optimal cut-off value of 0.99, the model demonstrated a sensitivity of 62.7%, a specificity of 69.2%, and an overall accuracy of 65.2%. The Youden index was 0.319, indicating that while the ratio possesses moderate discriminatory efficacy, it serves as a viable indicator for hepatic decompensation.

#### Differentiation between DC and ACLF stages

As the disease progressed to more severe stages, the E/Er Ratio further diverged between the DC and ACLF (*n* = 64) groups. The median ratio in the ACLF group (1.59) was significantly higher than that in the DC group (1.19). ROC analysis yielded an AUC of 0.651 (95% CI: 0.584–0.720) (Fig. 6B). At the optimal cut-off value of 1.44, the model achieved a sensitivity of 59.4%, a specificity of 68.1%, and an overall accuracy of 66.4%, with a Youden index of 0.275. These findings suggest that the E/Er Ratio maintains a moderate but significant discriminatory value in predicting the transition from decompensated cirrhosis to ACLF.

#### Conclusion of marker performance

The E/Er Ratio exhibits a progressive upward trend as liver disease advances from the compensated stage to the decompensated stage and ultimately to ACLF. It demonstrates statistically significant discriminatory power at two critical progression nodes (CC vs. DC and DC vs. ACLF), with AUCs of 0.659 and 0.651, respectively. As a non-invasive, microbiome-derived marker, the E/Er Ratio provides a potential auxiliary biomarker for the dynamic risk assessment of liver disease progression. Its clinical utility is likely to be further enhanced when integrated into multivariate predictive models that include established clinical parameters.

## Discussion

This study represents a systematic effort to delineate the dynamic evolution of the gut microbiota throughout the entire clinical spectrum of CLD. By analyzing a large cohort of 947 patients, we demonstrated that the progression from CLD to ACLF is characterized by a “dual-track” dysbiosis: the progressive loss of homeostatic, butyrate-producing commensals and the opportunistic overgrowth of pro-inflammatory pathogens.

### The pathophysiological significance of the E/Er ratio

A key finding is the pathophysiological significance of the *Enterococcus/Eubacterium rectale* (E/Er) Ratio. This ratio increased markedly with disease severity, serving as a microbial indicator of gut-liver axis dysfunction.

*Enterococcus* has emerged as a key “pathobiont” in liver disease [13, 14]. Beyond its correlation with systemic inflammation, certain strains of *Enterococcus* faecalis are known to secrete cytolysin, a pore-forming toxin that directly triggers hepatocyte lysis and has been clinically linked to increased mortality in alcoholic hepatitis [15]. Our findings suggest that this “Enterococcal bloom” is not limited to alcoholic etiology but is a common feature of advanced cirrhosis and ACLF, potentially driven by the depletion of bile acids which normally inhibit the overgrowth of Gram-positive cocci [16].

Conversely, *Eubacterium rectale* is a cornerstone of the “anti-inflammatory” microbial guild [17]. As a major producer of butyrate, it provides the primary energy source for colonocytes and induces the expression of tight junction proteins (e.g., zonula occludens-1 and occludin), which are essential for maintaining the physical integrity of the intestinal barrier [18, 19]. The “Sustained-Decline” pattern of *Eubacterium rectale* observed in our study suggests a chronic state of “starvation” and increased permeability of the intestinal epithelium (the “leaky gut”). This allows the translocation of endotoxins and other Pathogen-Associated Molecular Patterns (PAMPs) into the portal vein, where they activate Kupffer cells via TLR4 signaling, thereby fueling the hepatic necro-inflammatory cascade [20]. Regarding the cross-taxonomic nature of this ratio, *Enterococcus* was targeted at the genus level because multiple pathogenic species within this genus (e.g., E. faecalis, E. faecium) share clinically relevant virulence traits such as cytolysin production and translocation capacity. In contrast, *Eubacterium rectale* was chosen at the species level for its highly specialized and well-characterized butyrate-producing function, which is not uniformly present across the broader Eubacterium genus. This function-driven, cross-taxonomic comparison enhances the biological interpretability and clinical translatability of the E/Er Ratio as a qPCR-based biomarker.

### Mechanistic insight into the “four dynamic patterns”

The classification of 10 dominant gut microbiota into four dynamic patterns provides a roadmap for understanding microbial succession during liver disease progression.

The “Progressive Depletion” pattern of *Faecalibacterium prausnitzii* and *Eubacterium rectale* is of particular clinical significance. These two species represent the most potent butyrate-producers in the human gut, and their continuous decline from the earliest disease stage (CLD) onwards indicates that the erosion of protective, barrier-enhancing functions begins long before overt clinical decompensation. The sustained loss of these anti-inflammatory commensals likely contributes to the progressive impairment of intestinal barrier integrity, facilitating the translocation of pathogen-associated molecular patterns (PAMPs) and perpetuating hepatic necro-inflammation. This early depletion may serve as a subclinical sentinel of gut-liver axis disruption and offers a potential window for preventive interventions aimed at restoring butyrate-producing capacity.

The “decline-then-rise” pattern of *Bifidobacterium* and *Atopobium* cluster is particularly intriguing. While often considered beneficial, their late-stage rebound in DC and ACLF might reflect a compensatory but ineffective response to the extreme metabolic shifts in the gut environment, or perhaps the expansion of specific sub-strains that are better adapted to high-inflammatory conditions.

The “fluctuating-rise” of *Lactobacillus* aligns with observations in other critical illnesses, where certain lactic acid bacteria expand in response to the depletion of other commensals, although they may not fully replace the lost metabolic functions of butyrate producers.

The “rise-then-decline” pattern of *Bacteroides* and *Clostridium leptum*, characterized by a relative increase during the transition from CLD to CC followed by a precipitous decline in the ACLF stage, likely reflects an initial compensatory microbial remodelling that eventually collapses as the gut environment deteriorates. The early rise in these microbiota may represent a host-adaptive response to maintain metabolic homeostasis, but their subsequent decline signals the exhaustion of this compensatory capacity and the onset of irreversible gut ecosystem failure in end-stage liver disease.

### Machine learning for precision hepatology

#### Mechanistic insights and robustness of microbial biomarkers amidst clinical confounders in advanced liver disease

Notably, while the Random Forest model for the DC-to-ACLF transition achieved the highest absolute performance (AUC = 0.961), the incremental gain in AUC upon adding microbial features to clinical parameters was relatively modest compared to the clinical-only model. This phenomenon can be attributed to the ‘ceiling effect’ in end-stage liver failure, where extreme laboratory abnormalities (e.g., profound hyperbilirubinemia and coagulopathy) already provide near-maximal discriminatory power [21].

However, the inclusion of microbiome-derived indices—specifically the Specific-Butyrate-to-Total ratio—remains highly significant for several reasons. First, it confirms that the catastrophic collapse of the hepatic system is closely intertwined with a systemic failure of the gut ecosystem, providing a mechanistic ‘gut-centric’ explanation for the observed clinical phenotype. Second, unlike biochemical markers which are outcomes of organ failure, the progressive depletion of butyrate-producers may precede the peak of clinical symptoms, offering a wider window for early warning. Finally, the high weight of microbial features in the model, despite the modest AUC increment, underscores their potential as independent biological indicators that remain stable even when clinical parameters are confounded by acute medical interventions (e.g., albumin infusion or plasma exchange).

The moderate AUC (≈ 0.65) of the E/Er Ratio as a standalone marker for CC and DC suggests that while it is a significant biological indicator, the complex nature of liver decompensation requires a multi-dimensional approach. However, the non-invasive nature of this qPCR-based ratio makes it a highly attractive candidate for longitudinal monitoring, providing a “liquid biopsy” of the gut ecosystem that can be performed more frequently and safely than a liver biopsy or even repeated endoscopy.

#### The “sentinel” role of microbial biomarkers: explaining the superiority of early-stage predictive performance

This study reveals a critical phenomenon: while traditional clinical biochemical parameters—such as TBil, Alb, and PT—are highly effective in predicting terminal liver failure (e.g., the progression from DC to ACLF) due to their direct reflection of organ synthetic and metabolic collapse, microbiome features show superior sensitivity in capturing earlier disease tipping points, specifically the transition from CLD to CC/DC. In particular, the *Enterococcus/Eubacterium rectale* (E/ER) ratio stands out as a key indicator. This stage-specific variation in discriminatory performance can be elucidated through two dimensions: the physiological signaling delay of the “gut-liver axis” and the clinical heterogeneity across different disease cohorts.

Firstly, the fundamental cause lies in the physiological signaling delay inherent to the gut-liver axis. During the early stages of liver disease, the liver’s compensatory capacity remains relatively intact; consequently, conventional serological markers (e.g., albumin and bilirubin) often persist within normal or borderline ranges, lacking the dynamic range necessary to sensitively indicate early injury. In contrast, the intestinal microecosystem acts as a “front-line sentinel” for systemic inflammatory states and changes in bile acid metabolism, making it exquisitely sensitive to liver-derived pathophysiological shifts. This study observed that the progressive depletion of protective butyrate-producing bacteria occurs well before serological markers become significantly abnormal. This depletion directly reflects early damage to the intestinal mucosal barrier and the loss of local immunomodulatory functions [22]. Concurrently, the proliferation of opportunistic pathogens serves as an advanced warning signal for increased risks of bacterial translocation [5, 23]. By integrating these subtle but directional microbial shifts via machine learning models, we can identify “pre-clinical” or “sub-clinical” progression states missed by traditional markers, thereby securing a critical time window for intervention.

Secondly, the clinical heterogeneity of the research cohorts amplifies these biological differences. The CLD patient cohort included in this study exhibits higher homogeneity; these patients have not yet developed severe complications and are less affected by confounding medications (such as diuretics and antibiotics). Thus, the observed dysbiosis is more likely specifically attributable to the progression of the liver disease itself. Conversely, patients with DC and ACLF commonly present with multiple complications, including ascites, spontaneous bacterial peritonitis, and renal injury. The corresponding therapeutic measures significantly and heterogeneously reshape the intestinal microenvironment, inevitably increasing the difficulty of extracting universal, robust microbial predictive signals from such complex “noise” during these advanced stages.

In summary, the superior performance of microbial biomarkers such as the E/Er Ratio in early-stage models does not represent an absolute decay in their predictive power; rather, their core value lies in the early warning of disease progression. They function as “distal amplifiers” and “sensitive sensors” of hepatic microenvironmental deterioration, providing vital risk signals even before clinical biochemical indicators have “spoken.” This finding not only deepens our understanding of the chronological dialogue within the gut-liver axis but also strongly suggests that integrating microbiome information into liver disease management frameworks holds significant translational medicine value for achieving true early risk stratification and preventive intervention.

### Strengths and limitations

#### Strengths

The strength of this study lies in its large-scale, real-world cohort and the focus on 10 clinically actionable microbiota. Our results support a shift from a “liver-centric” to a “gut-liver” therapeutic strategy.

Early-Stage Intervention: In CLD and CC, therapies should focus on “fortifying the barrier” through high-fiber diets, prebiotics, or probiotics containing *Eubacterium rectale* and *Faecalibacterium prausnitzii* to prevent the initial transition to decompensation [24].

Late-Stage Intervention: In DC and ACLF, the focus must shift to “decontaminating the gut.” The proliferation of *Enterococcus* and *Enterobacterium* suggests that targeted non-absorbable antibiotics (like Rifaximin) or even Fecal Microbiota Transplantation (FMT) might be necessary to break the vicious cycle of dysbiosis and systemic inflammation [25].

#### Limitations

However, several limitations must be acknowledged. First, the retrospective design precludes causal inference regarding whether dysbiosis drives or results from liver failure. Second, the cross-sectional design with single admission-time sampling precludes true temporal modeling; future prospective studies with serial sampling should explore temporal deep learning architectures (e.g., RNNs, LSTMs). Third, while qPCR is highly accurate for quantification, it lacks the broad discovery potential of 16 S rRNA or Metagenomic Shotgun Sequencing, which could reveal functional gene changes. Fourth, the study did not account for variations in diet or prior medication use (beyond the 4-week exclusion period), which are known modulators of the gut microbiome. Fifth, the absence of a healthy control group limits our ability to determine the baseline degree of dysbiosis in the CLD cohort; our findings therefore reflect disease progression relative to the CLD state rather than from a healthy baseline. Future studies incorporating healthy controls are warranted to establish the full trajectory of microbiome alteration from health to liver failure.

In conclusion, chronic liver disease progression is intrinsically linked to a systemic reorganization of the gut microbiota. The E/Er Ratio and the associated machine learning models provide a novel, non-invasive framework for predicting disease turning points. These findings not only enhance our understanding of the gut-liver axis but also provide a scientific rationale for the development of personalized, gut-targeted therapies to improve the prognosis of patients with chronic liver disease.

## Conclusion

In summary, this study systematically delineates the dynamic evolution of the gut microbiota throughout the entire progression of chronic liver disease, from initial injury to ACLF. Our findings reveal a profound and structured realignment of the gut ecosystem, characterized by the sustained depletion of protective butyrate-producing commensals—notably *Eubacterium rectale* and *Faecalibacterium prausnitzii*—and the explosive late-stage proliferation of opportunistic pathogens such as *Enterococcus*.

Through machine learning analysis, we demonstrated that integrating microbiome-derived indices, such as the Specific-Butyrate-to-Total ratio, with conventional clinical parameters significantly enhances the robustness of disease progression models. Although clinical indicators remain dominant predictors in the late stages of liver failure, the gut microbiota provides indispensable biological insights into the systemic collapse of the gut-liver axis and offers a potentially more stable “sentinel” signal for early warning.

Specifically, the E/Er Ratio emerged as a promising, non-invasive biomarker capable of identifying critical transition points, including the onset of decompensation and the progression to ACLF. Collectively, our findings advocate for a paradigm shift from a “liver-centric” to a “gut-liver axis” perspective in understanding disease progression, providing a strong scientific rationale for future personalized, gut-targeted therapeutic strategies. Ultimately, early monitoring and modulation of the intestinal microecology may pave the way for precise interventions to delay liver disease progression and improve patient outcomes.

## Supplementary Information

Below is the link to the electronic supplementary material.

Supplementary Material 1

Supplementary Material 2

Supplementary Material 3

Supplementary Material 4

Supplementary Material 5

Supplementary Material 6

## Data Availability

Due to the privacy policy, the datasets analyzed in this study are not publicly available, but they are available from the corresponding author upon reasonable request. The analysis code is available at: https://github.com/mahuahuogun/liver-microbiome-machine-learning.

## Abbreviations

ACLF: Acute-on-Chronic Liver Failure
Alb: Albumin
ALP: Alkaline Phosphatase
ALT: Alanine Aminotransferase
apoA1: Apolipoprotein A1
APTT: Activated Partial Thromboplastin Time
AST: Aspartate Aminotransferase
AUC: Area Under the Curve
BUN: Blood Urea Nitrogen
CC: Compensated Cirrhosis
ChE: Cholinesterase
CLD: Chronic Liver Disease
CRP: C-Reactive Protein
DBil: Direct Bilirubin
DC: Decompensated Cirrhosis
DD: D-dimer
E/Er Ratio: Enterococcus/Eubacterium rectale Ratio
eGFR: Estimated Glomerular Filtration Rate
FDR: False Discovery Rate
FIB: Fibrinogen
HDL: High-Density Lipoprotein
hs-CRP: High-sensitivity C-Reactive Protein
INR: International Normalized Ratio
IQR: Interquartile Range
LDL: Low-Density Lipoprotein
NLR: Neutrophil-to-Lymphocyte Ratio
NPAR: Neutrophil Percentage-to-Albumin Ratio
PAMPs: Pathogen-Associated Molecular Patterns
PLT: Platelet Count
PT: Prothrombin Time
RF: Random Forest
ROC: Receiver Operating Characteristic
SCFA: Short-Chain Fatty Acid
SII: Systemic Immune-Inflammation Index
TBA: Total Bile Acid
TBil: Total Bilirubin
TC: Total Cholesterol
TG: Triglyceride
TT: Thrombin Time
UA: Uric Acid
WBC: White Blood Cell Count
